# Application of 3D Printing to Design and Manufacture Pancreatic Duct Stent and Animal Experiments

**DOI:** 10.3390/bioengineering11101004

**Published:** 2024-10-08

**Authors:** Fu Xiang, Chenhui Yao, Guoxin Guan, Fuwen Luo

**Affiliations:** 1 The Second Affiliated Hospital of Dalian Medical University, Dalian 116021, China; dyfy-xiangfu@outlook.com (F.X.); dlggx1978@163.com (G.G.); 2 The First Affiliated Hospital of Dalian Medical University, Dalian 116021, China; gavinyaoch@126.com

**Keywords:** postoperative pancreatic fistula, pancreaticoduodenectomy, pancreatojejunostomy, 3D printing, biodegradable stent

## Abstract

**Objective:** Postoperative pancreatic fistula (POPF) is a common and challenging complication following pancreaticoduodenectomy (PD), occurring in 2% to 46% of cases. Despite various pancreaticojejunostomy techniques, an effective method to prevent POPF has not been established. This study aimed to develop and evaluate a novel 3D-printed biodegradable pancreatic duct stent to simplify the surgical process of pancreaticojejunostomy, reduce anastomotic complexity, and minimize postoperative complications. **Methods:** Data from 32 patients undergoing total laparoscopic pancreaticoduodenectomy were utilized. Preoperative CT scans were transformed into 3D reconstructions to guide the design and printing of customized stents using polylactic acid (PLA). The stents were assessed for mechanical integrity, surface texture, and thermal stability. Animal experiments were conducted on 16 mini pigs, with the experimental group receiving the novel stent and the control group receiving traditional silicone stents. **Results:** The 3D-printed stents demonstrated accurate dimensional replication and mechanical reliability. In the animal experiments, the experimental group showed no significant difference in postoperative complications compared to the control group. At 4 weeks post-surgery, CT scans revealed well-healed anastomoses in both groups, with no significant inflammation or other complications. Histological examination and 3D reconstruction models confirmed good healing and device positioning in the experimental group. **Conclusion:** The 3D-printed biodegradable pancreatic duct stent offers a promising solution for pancreaticojejunostomy, with comparable safety and efficacy to traditional methods. Further research is needed to validate its clinical application.

## 1. Introduction

Postoperative pancreatic fistula (POPF) represents a formidable challenge following pancreaticoduodenectomy (PD), with an incidence that varies from 2% to 46% [1,2,3]. Despite the proliferation of over 30 pancreaticojejunostomy techniques in clinical practice, a definitive method to prevent POPF remains elusive [4]. While numerous studies have heralded the use of stents in reducing the incidence of clinically relevant POPF [5,6,7,8], a counter-narrative suggests no significant difference in surgical outcomes with or without stent use [9,10,11]. However, internal stent drainage has been associated with a lower rate of biochemical fistula [10]. The clinical mainstream continues to favor the use of pancreatic duct stents, predominantly in two forms: internal and external drainage. External drainage, often employed in open pancreaticoduodenectomy (OPD), aims to reduce the risk of stent displacement and the potential for it to spontaneously detach into the intestinal lumen [12]. Conversely, internal drainage is frequently utilized in laparoscopic pancreaticoduodenectomy (LPD). Despite the advantages, the removal of external drainage stents may lead to mechanical injury at the anastomotic site, resulting in pancreatitis or pancreatic duct obstruction [13]. Moreover, the substantial loss of pancreatic juice through external drainage can precipitate risks such as electrolyte imbalances, gastrointestinal dysfunction, and malnutrition [11]. These drawbacks have led some surgeons to prefer internal stent drainage due to its potential to minimize digestive fluid loss and eliminate the need for stent removal.

**Figure 1:**
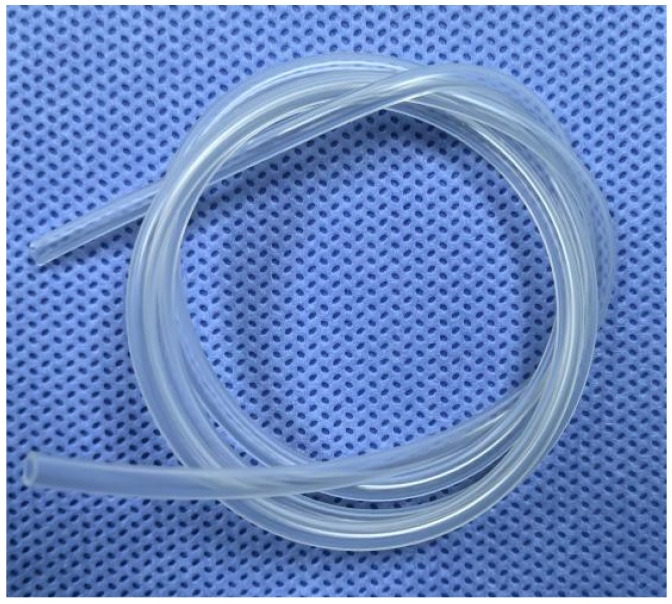
Traditional silicone internal drainage stent tube.

The conventional internal drainage stent tubes, typically made of silicone and cylindrical in shape (as depicted in Figure 1), are limited in size options. A mismatch in size between the stent tube and the pancreatic duct can lead to complications; a stent tube that is significantly smaller than the duct can leave an excessive gap, increasing the risk of fistula, while a closely matching size can make suturing more challenging. The author has encountered a clinical case where internal stent tube displacement led to small bowel perforation due to the tube end pressing against the intestinal wall. A follow-up of over 800 LPD cases by Professor Peng Bing’s team at West China Hospital revealed that pancreatic stent use could lead to pancreatic duct stones, stent displacement, obstruction of branch pancreatic ducts, pancreatitis, and intestinal perforation [10]. The alternative of performing a pancreatic duct-mucosa pancreaticojejunostomy without a stent tube is exceedingly difficult and is only conducted in a few major pancreatic centers. These considerations have spurred the need for a new type of degradable pancreatic duct stent tube that meets surgical requirements, reduces procedural complexity, and minimizes surgical complications, marking a novel direction for research.

The incidence of POPF is influenced by multiple factors, with the anastomosis technique being an independent risk factor [6]. The consensus is that reducing the number of sutures through the pancreas and reinforcing the anastomotic site can significantly lower the risk of POPF, which is closely tied to the surgeon’s skill and experience [8,9]. The advent of 3D printing has introduced unique advantages in clinical applications, particularly in the context of complex anatomical structures like the pancreas. Three-dimensional printing, emerging in the 1970s, leverages digital models from medical imaging to construct objects layer by layer using various materials, including metals, ceramics, resins, and bio-polymers. This technology has seen rapid growth in fields such as tissue engineering, digital medicine, and medical education [10,11,12]. The pancreas, with its complex and variable shape, is challenging to represent in two dimensions, making 3D reconstruction a novel approach for studying abdominal organs and addressing surgical challenges [13]. The precision, speed, and customization offered by 3D printing are particularly advantageous in assisting surgical procedures and fabricating personalized medical devices [14]. Fused deposition modeling (FDM), a common 3D printing process, utilizes bio-polymers such as polylactic acid, polycaprolactone, polycarbonate, and polyamide, which are biodegradable and characterized by low cost, high mechanical strength, and minimal environmental impact. These bio-absorbable materials are advantageous for their low toxicity, minimal foreign body reactions, and negligible long-term impact on patients, aligning with the future direction of medical product development.

The goal of this research is to develop a novel pancreatic duct stent using 3D printing technology. This stent is designed to streamline the surgical process of pancreaticojejunostomy, reduce the complexity of the anastomosis, and ultimately decrease the incidence of postoperative complications, specifically pancreatic fistulas.

## 2. Method

### 2.1. Patient Selection and Data Collection

This investigation encompassed a meticulous data collection from 32 patients who underwent total laparoscopic pancreaticoduodenectomy at the Department of General Surgery, First Affiliated Hospital of Dalian Medical University, Jinpu Campus, over the period from March 2022 to August 2024. Informed consent was secured from all participants at the outset of the study. As part of the preoperative assessment, each patient underwent a computed tomography (CT) scan of the upper abdomen using an OPTIMA CT660 scanner (GE Healthcare, Chicago, IL, USA), incorporating plain and contrast-enhanced triple-phase thin-slice imaging. This study complies with the Declaration of Helsinki and has been approved by the Ethics Committee of the First Affiliated Hospital of Dalian Medical University (PJ-KS-KY-2024-443).

The patient selection process was governed by stringent inclusion and exclusion criteria. This study included patients aged between 18 and 75 years and exhibiting ultrasound indications of tumors in the pancreatic head, lower common bile duct, papilla, or ampulla, which necessitated surgical intervention. Patients were excluded based on an age above 75 years, the presence of tumors in the pancreatic body or tail, evidence of acute or chronic pancreatitis, the encasement of major arteries by tumors exceeding 180 degrees, or the presence of distant metastatic disease.

### 2.2. CT Scanning and 3D Reconstruction

**Figure 2:**
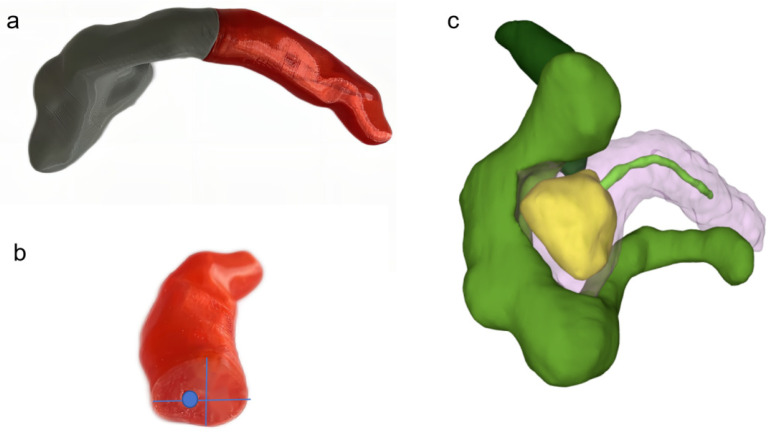
Three-dimensional reconstruction: (**a**) 3D modeling of pancreatic duct; (**b**) dimensional measurement; (**c**) 3D reconstruction of pancreatic head cancer.

Preoperatively, patients underwent an abdominal CT scan with plain and contrast-enhanced triple-phase imaging using a high-resolution scanner (OPTIMA CT660 scanner, GE Healthcare, USA). The DICOM format data obtained were then processed using Materialise Mimics software (Materialise, version 21.0 Belgium) to convert the thin-slice CT data into three-dimensional reconstructions. These reconstructions served as a detailed database for pancreatic imaging, creating accurate anatomical representations. Advanced workstations were employed for further data processing, utilizing Materialise’s 3-matic Medical software (Materialise, version 13.0 Belgium) for the initial modeling and texturing of pancreatic structures. Subsequent refinement and detail enhancement were performed using 3D Max, followed by final adjustments in Mimics to ensure the accuracy and fidelity of the 3D models. We meticulously measured the anteroposterior and superoinferior diameters of the pancreatic neck as well as the diameter of the pancreatic duct, ensuring the dimensions were captured with precision for the 3D-printed stent design (Figure 2).

Intraoperative measurements were compared with preoperative imaging data to validate the 3D models’ accuracy, ensuring their reliability in surgical guidance and anatomical model printing.

### 2.3. Design and 3D Printing of the Pancreatic Duct Stent

**Figure 3:**
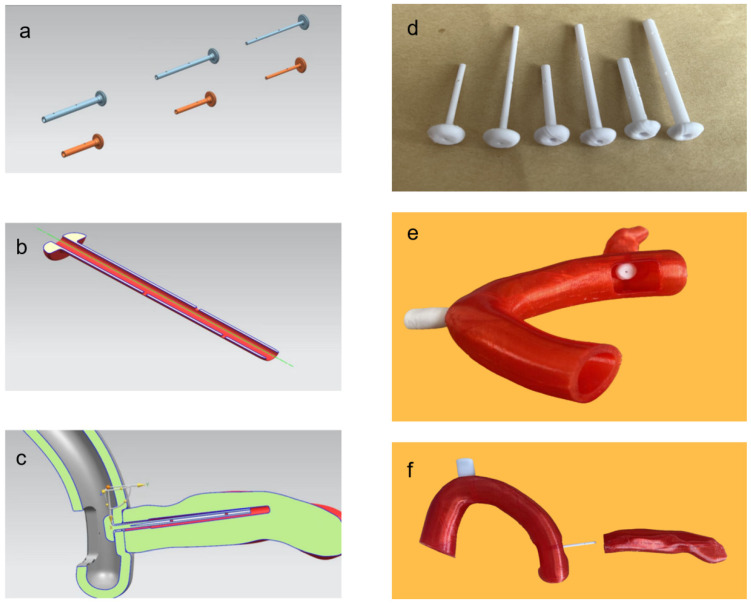
Design of the pancreatic duct stent: (**a**) design of novel pancreatic duct stent; (**b**) internal structure of the stent; (**c**) schematic diagram of the placement of the novel stent; (**d**) pancreatic duct stent finished product; (**e**) side view of pancreaticojejunostomy; (**f**) front view of pancreaticojejunostomy.

The 3D reconstructions guided the 3D printing process, replicating the patient’s pancreas at a 1:1 scale using a Stratasys Fortus 400 mc 3D printer (Stratasys, Eden Prairie, MN, USA). At the level of the pancreatic neck, a transverse cut was made along the left margin of the portal vein to facilitate detailed examination. For the design and fabrication of the novel pancreatic duct stent, Siemens NX 12.0 software (Siemens PLM Software, version 12.0, USA) was applied. The stent was printed using a Voron Trident 3D filament printer (Dalian Join Industry Technology Co., Ltd., Dalian, China) with medical-grade polylactic acid (PLA) material (Zhejiang Hisun Biomaterials Co., Ltd., Zhejiang, China), which is a high-molecular-weight PLA with a weight-average molecular weight (Mw) of approximately 200,000 to 300,000 g/mol. The 3D printing was completed at Dalian Join Industry Technology Co., Ltd. (China), where we designed a novel pancreatic duct stent tailored to patient-specific anatomical variations. Manufactured from polylactic acid (PLA) for biocompatibility and biodegradability, the stent featured a 0.5 mm wall thickness and varied outer diameters (2 mm, 3 mm, 4 mm) and lengths (3 cm, 5 cm) to fit diverse pancreatic duct sizes and anastomotic requirements (Figure 3). Strategic lateral holes on the pancreatic end and a 1 cm-diameter disc at the jejunal end were integrated for optimal fluid drainage and tissue integration. The diameter of the PLA filament was 1.75 mm, the printing temperature was 210°, the printing layer thickness was 0.1 mm, and the printing speed was 50 mm/s.

After printing, the stents were first subjected to a thorough cleaning process to remove any residual printing material or support structures. This was followed by a sterilization procedure using ethylene oxide, which is effective in eliminating bacteria and other pathogens without compromising the material’s integrity.

Barium sulfate was incorporated into the PLA filament at a concentration of 15% by weight to impart radiopaque properties to the stent. During the 3D printing process on a Stratasys Fortus 400 mc 3D printer, the filament was extruded in such a way that a higher concentration of barium sulfate was localized at the stent’s head, facilitating non-invasive postoperative X-ray monitoring. Post-printing, each stent underwent a comprehensive quality assurance process to ensure it met clinical standards. The dimensional accuracy was verified using a vernier caliper and confirmed through coordinate measurement machine (CMM) technology. The structural integrity was assessed via mechanical testing on an electronic universal testing machine (UTM-1432, Chengde Jinjian Testing Instrument Co., Ltd., Chengde, China) and microstructural examination with a ZEISS field emission scanning electron microscope. The uniform distribution of the radiopaque material was evaluated using X-ray imaging and an X-ray diffractometer (MiniFlex600, Nippon Rigaku Co., Ltd., Tokyo, Japan). Thermogravimetric analysis (TGA) confirmed the thermal stability of the PLA and radiopaque components. The chemical composition was analyzed with an infrared spectrometer (TENSOR 27, Bruker Optics, Billerica, MA, USA) to ensure material purity. All data were documented and analyzed using Origin software (version 2023), and only stents that passed all inspections, including a final review by a quality assurance officer, were approved for clinical application.

### 2.4. Material Characterization of 3D-Printed Stents

The mechanical integrity of the 3D-printed stents was assessed through a series of standardized tests to evaluate their structural reliability. The tensile strength and elastic modulus, which are critical parameters for understanding how a material responds to applied forces, were measured according to the ISO 527-1:2012 standards. For these tests, stent samples were prepared and gripped securely in an electronic universal testing machine (UTM-1432, Chengde Jinjian Testing Instruments Co., Ltd., Chengde, China). The samples were subjected to a tensile load at a controlled rate of 2 mm/min until failure occurred. The load cell of the UTM ensured accurate measurement of the force applied, and the crosshead displacement was recorded to track the elongation of the samples. The tensile strength was calculated by dividing the maximum load at break by the initial cross-sectional area of the stent. The elastic modulus was determined from the slope of the stress–strain curve in the linear elastic region. The surface microstructure and texture were analyzed using an Olympus BX51 microscope (Olympus Corporation, Tokyo, Japan) to assess their impact on tissue integration. X-ray diffraction (XRD) was performed with a Rigaku MiniFlex 600 benchtop XRD system (Rigaku Corporation, Tokyo Japan) to confirm the crystallinity and molecular structure of the PLA material, ensuring quality and purity. Thermogravimetric analysis (TGA) was conducted on a Mettler Toledo TGA/DSC 1 STARe system (Mettler Toledo, Zurich, Switzerland) to determine the thermal stability of the PLA, crucial for its in vivo degradation profile.

### 2.5. Animal Experiments

**Figure 4:**
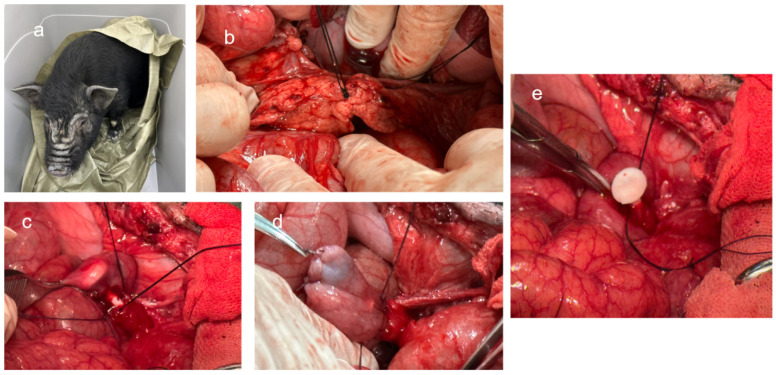
Animal experiment pictures: (**a**) Tibetan miniature pig; (**b**) pancreatic neck ligation in the first stage of surgery; (**c**) pancreatic duct stent placement in the second stage of surgery; (**d**) pancreaticojejunostomy completed in the second stage of surgery; (**e**) good matching of the stent tube in the second stage of surgery.

This study involved the use of 16 Tibetan miniature pigs for the in vivo evaluation of a novel pancreatic duct stent. The animals were randomly assigned to either the experimental group or the control group, each comprising eight mini pigs with an equal sex distribution (Figure 4). The experimental group was subjected to pancreaticojejunostomy using the novel stent, while the control group received conventional silicone stents. This study has been approved by the Experimental Animal Ethics Committee of Dalian Medical University (AEE23017).

The anesthetic method for Tibetan miniature pigs included fasting from 8 PM the night before surgery, with intraoperative fluid replenishment. Pre-anesthetic medication involved an intramuscular injection of atropine sulfate at 0.02 mL/kg to reduce glandular secretion. Anesthesia was induced with an intramuscular injection of the combined anesthetic agent Telazol (tiletamine and zolazepam) at 0.1 mL/kg. After satisfactory anesthesia, endotracheal intubation was performed with balloon-assisted ventilation. The vital signs were monitored using electrocardiography and pulse oximetry to ensure surgical safety, and electrocautery was employed for hemostasis to reduce blood loss and expedite the surgical process.

The first-stage surgery involved the creation of a pancreas duct dilation model through ligation of the pancreatic neck. This step was critical due to the narrow diameter of the pancreatic duct in mini pigs, which could complicate direct pancreaticojejunostomy and potentially lead to surgical failure. Dilation of the duct better simulated the pathological state of disease, thus requiring preliminary expansion of the main pancreatic duct. Under anesthesia, a midline abdominal incision was made, the pancreas was exposed, and the neck was carefully ligated with non-absorbable silk sutures. The intestines were repositioned, and the incision was meticulously closed in layers. Postoperatively, a full liquid diet was initiated six hours after the first surgery, transitioning to a semi-liquid diet 24 h post-op.

The second-stage surgery was performed 2 to 4 weeks after the initial procedure, following the preparation and sterilization of the stents. For the second surgery, fluid replenishment was continued within the first 24 h, followed by a full liquid diet if no complications arose, and then a semi-liquid diet for one week. Blood samples were taken 2 days preoperatively and compared against the baseline values; only animals with parameters within the reference range were included in the surgery. CT scans were conducted to ascertain the pancreatic duct diameter, and 3D-printed stents were tailored accordingly. The experimental group received the innovative stent, while the control group was fitted with silicone stents. The original incision was reopened, adhesions were lysed, and the pancreas was transected. A Roux-en-Y pancreaticojejunostomy was executed with the jejunum approximately 20 cm from the pancreatic head. The novel stent was carefully inserted into the main pancreatic duct, and the jejunum was anastomosed to the pancreatic stump. The anastomosis site was inspected for bleeding, and a drainage tube was placed prior to closure. Postoperative care entailed daily monitoring of the animals’ conditions and periodic blood collection for ELISA to detect inflammatory cytokines IL-6 and IL-10. CT imaging was utilized to ascertain the positioning of the stent, and potential complications, such as pancreatic fistula, intestinal obstruction, peritoneal infection, postoperative bleeding, and acute pancreatitis, were vigilantly monitored. In cases of severe complications leading to a critical condition, euthanasia was performed. For the second surgery, fluid replenishment was continued within the first 24 h, followed by a full liquid diet if no complications arose, and then a semi-liquid diet for one week. We tailored the stent designs to match the diameters of the pancreatic ducts as measured from the preoperative imaging of the mini pigs. For the control group, we selected from two sizes of silicone stents that were available: 2 mm and 3 mm in diameter. In three instances where the pancreatic duct diameter was less than 2 mm—specifically, two cases in the experimental group with diameters of 1.9 mm each, and one case in the control group with a diameter of 1.8 mm—we took specific measures to ensure proper stent placement. For the experimental group, we adjusted the 3D printing parameters to directly print stents with a diameter of 1.9 mm. For the control group, we chose the 2 mm silicone stent and adopted a technique where we first sutured traction threads around the 1.8 mm duct. We then applied a small amount of sterile petrolatum to the surface of the silicone stent to facilitate its gentle insertion. For all other animals in the study, the stent placement was executed smoothly without any complications.

The third-stage surgery was executed two months following the second procedure. Blood samples were collected for ELISA to evaluate cytokines IL-6 and IL-10. After euthanasia, the anastomotic site was examined, and the pancreaticojejunostomy segment was excised for histopathological examination using immunohistochemistry and hematoxylin-eosin staining to assess inflammatory responses. A comparative analysis of local and systemic inflammatory responses, surgical time, anastomosis duration, and postoperative complications between the two groups was conducted to assess the safety and benefits of the novel stent.

The euthanasia method for Tibetan miniature pigs involved administering the anesthetic ketamine via intramuscular injection at a dosage of 5 mg/kg as a single, sufficient dose. Once the desired level of sedation was achieved, the pigs were humanely euthanized using the method of arterial bloodletting.

### 2.6. Statistical Methods

Statistical analysis was conducted using SPSS software (version 25.0) for Windows. The data were tested for normality with the Shapiro–Wilk test, and parametric (Student’s *t*-test) or non-parametric (Mann–Whitney U test) methods were applied accordingly to compare continuous variables between groups. The categorical data were analyzed using the Chi-square or Fisher’s exact test. Repeated measures were assessed by ANOVA, and correlations were determined using the Pearson or Spearman correlation coefficients. A *p*-value < 0.05 was considered statistically significant, ensuring the reliability of the results and their implications for the study’s conclusions.

## 3. Result

### 3.1. Results of CT Three-Dimensional Reconstruction Measurements

The three-dimensional reconstructions from CT scans allowed for precise measurements of the pancreatic neck’s anteroposterior diameter, superoinferior diameter, and the pancreatic duct’s diameter. A meticulous comparison was conducted between the preoperative imaging data and the actual intraoperative measurements of 32 patients (Table 1). The results demonstrate a high degree of accuracy in the three-dimensional reconstructions, with the maximum discrepancy being only 0.2 mm (Table 1). This close correlation between predicted and actual dimensions underscores the reliability of the CT reconstructions in guiding surgical procedures and the subsequent design of the customized 3D-printed pancreatic duct stent.

### 3.2. Material Characterization

**Figure 5:**
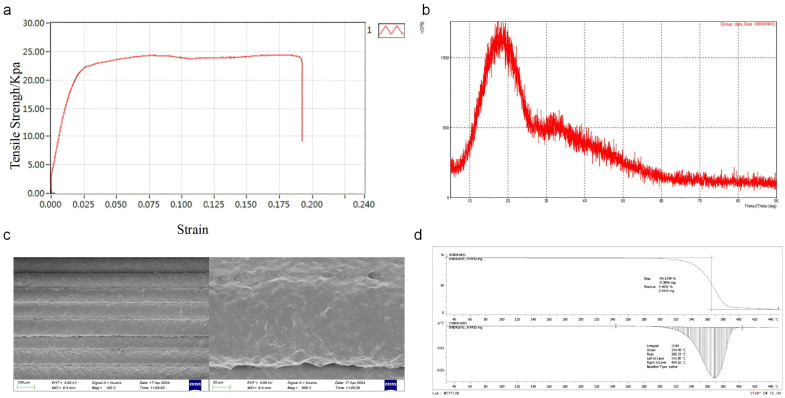
Material characterization: (**a**) tensile strength and strain of novel pancreatic duct stent; (**b**) XRD of novel pancreatic duct stent; (**c**) microscopic surface structure (100× and 500×); (**d**) thermogravimetric analysis.

Figure 5a illustrates the relationship between the tensile strength and strain, indicating that the tensile behavior of the sample went through three different stages: an elastic stage, a stable stage, and a fracture stage. This meets the requirements for the stability of the elastic deformation of the stent tube, indicating that 3D printing did not change the material’s ability to resist elastic deformation.

The XRD analysis presented in Figure 5b shows that the diffraction pattern of the 3D-printed pancreatic duct stent is consistent with that of the polylactic acid (PLA) filaments reported in the literature. This indicates that the 3D printing process did not significantly alter the structural properties of the PLA, preserving its crystallinity and molecular arrangement. The 3D-printed pancreatic duct stent exhibited XRD diffraction patterns consistent with those reported in the literature for polylactic acid filaments, indicating that the 3D printing process did not significantly affect the structure of polylactic acid.

The microscopic surface structure, as depicted in Figure 5c, reveals a regular lattice arrangement of the printed material with an even distribution across the plane. This uniformity is characteristic of layer-by-layer printing, where each layer is uniformly and tightly bonded, ensuring no gaps between layers, which is essential for the structural integrity of the stent.

The TGA of the 3D-printed PLA pancreatic duct stent (Figure 5d) demonstrated a melting temperature of 243.90 °C, a decomposition temperature of 369.33 °C, and an end-of-decomposition temperature at 404.16 °C. These thermal properties suggest that the material remained stable under physiological conditions, which is imperative for its performance as an implantable medical device.

### 3.3. Safety and Advantages of the Novel Pancreatic Duct Stent

The incidence of postoperative complications was closely monitored between the experimental and control groups of Tibetan miniature pigs. As depicted in Table 2, while both groups experienced similar occurrences of pancreatic fistula and acute pancreatitis, there were notable differences in other complications. The control group faced one instance of peritonitis and postoperative hemorrhage, culminating in one death due to a cascade of complications initiated by a pancreatic fistula. This underscores the importance of meticulous postoperative care and monitoring. Despite these events, the overall differences in postoperative complications between the experimental and control groups were not substantial.

The novel pancreatic duct stent demonstrated a significant advantage in terms of surgical efficiency. As shown in Table 3, the experimental group, utilizing the novel stent, had a reduced second surgery time and pancreaticojejunostomy time compared to the control group. This indicates that the novel stent can potentially streamline the surgical process, leading to a shorter duration under anesthesia and quicker recovery times for patients.

**Figure 6:**
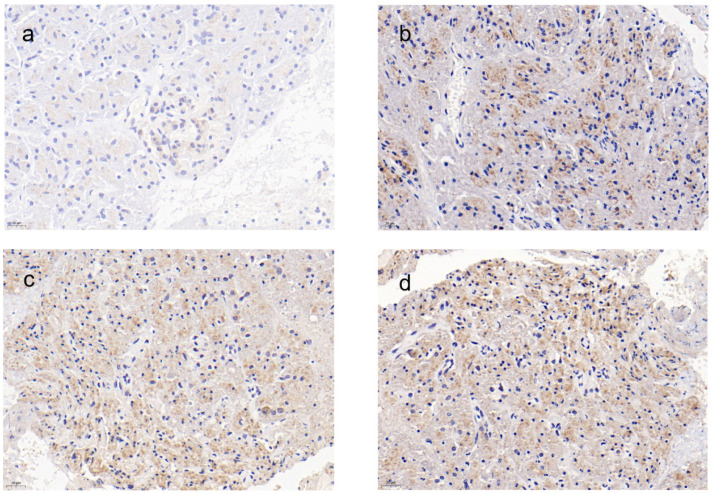
Immunohistochemical staining of IL-6 and IL-10 expression in anastomotic pancreatic tissue: (**a**) IL-6 immunohistochemistry of pancreaticojejunal anastomosis tissue in the experimental group, 20 μm; (**b**) IL-6 immunohistochemistry of pancreaticojejunal anastomosis tissue in the control group, 20 μm; (**c**) IL-10 immunohistochemistry of pancreaticojejunal anastomosis tissue in the experimental group, 20 μm; (**d**) IL-10 immunohistochemistry of pancreaticojejunal anastomosis tissue in the control group, 20 μm.

The immunohistochemical evaluation provided insights into the local inflammatory response at the anastomotic site (Figure 6). Both the experimental and control groups exhibited expressions of IL-6 and IL-10 in the pancreatic tissue cells at the anastomosis, with positive particles primarily located in the cytoplasm, appearing as brown granules. At 8 weeks post-pancreaticojejunostomy, the expression of IL-10 ranged from positive (+) to strongly positive (+++), while IL-6 showed positive (+) expression. Notably, when comparing the experimental group, which used the novel anastomotic device, with the control group, which utilized manual suturing, no statistically significant differences were observed (*p* > 0.05). This suggests that the novel pancreaticojejunostomy device did not increase the local inflammatory response at the anastomotic site, indicating its biocompatibility and potential to facilitate healing without exacerbating inflammation.

Two months after pancreaticojejunostomy, the comparative analysis of blood factor concentrations before the three-stage surgery showed that there was no significant difference in serum C-reactive protein (CRP) and procalcitonin (PCT) levels between the two groups, both of which remained at a low level. There were also no significant differences in the IL-6 and IL-10 levels (*p* > 0.05), as shown in Table 4.

**Figure 7:**
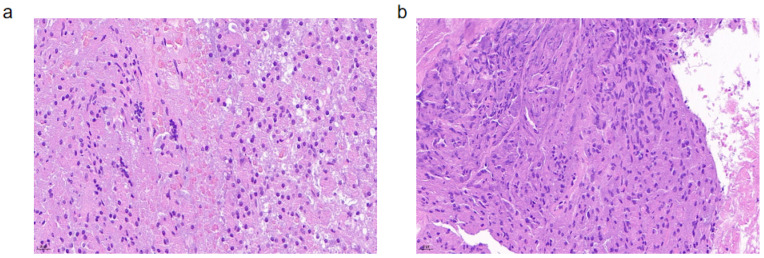
HE staining (20 μm) ((**a**) experimental group, (**b**) control group).

The anastomotic tissue was subjected to pathological HE staining, and microscopic observation revealed that the pancreatic tissue at the anastomosis site in both the experimental and control groups showed clear lobular structures without signs of extensive necrosis (Figure 7). In some areas, a small number of lobulated neutrophilic nuclei were observed. This indicates that the implantation of the new pancreatic stent in the experimental mini-pigs did not cause severe inflammatory reactions, and compared to the traditional silicone stent, it did not increase the occurrence of local inflammatory reactions at the anastomosis.

### 3.4. Gross Appearance of Anastomosis and Surgical Results

Following the lysis of abdominal adhesions and identification of the pancreatic neck along the ligation line from the initial surgery, the pancreas was transected distal to the ligation, anterior to the left edge of the portal vein. The experimental group underwent pancreaticojejunostomy using individually customized 3D-printed pancreaticojejunostomy devices, while the control group received traditional anastomosis with silicone pancreatic duct stent tubes. There were no significant differences in the gross appearance of the pancreaticojejunostomy sites between the two groups immediately after the anastomosis.

**Figure 8:**
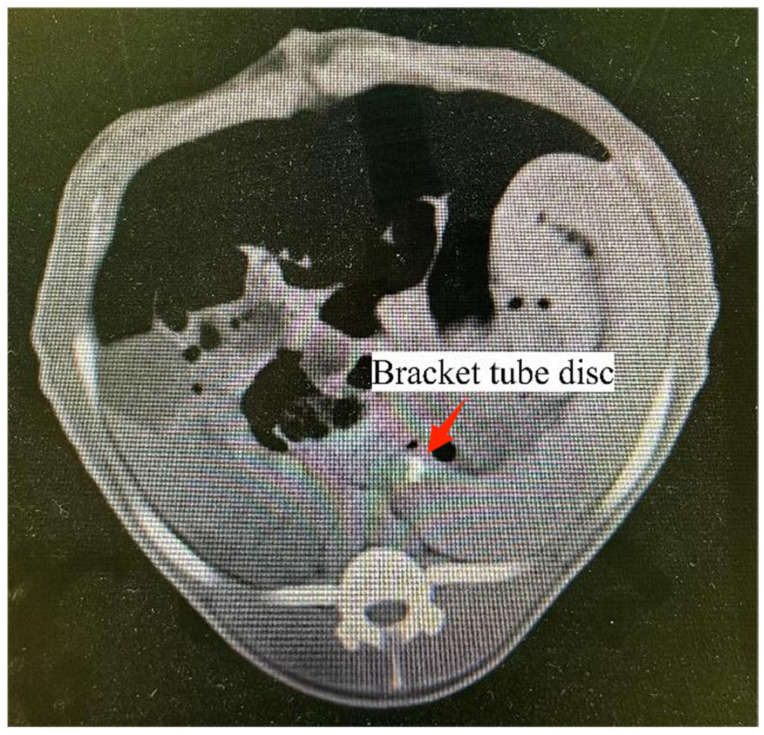
The novel stent was visualized in the second-stage postoperative follow-up CT scan. The stent was in a good position and was visualized because barium sulfate was added to the head of the stent tube.

Four weeks post-pancreaticojejunostomy, both the experimental and control groups underwent contrast-enhanced pancreatic CT scans. The imaging revealed well-healed anastomoses in both groups, with no significant effusion, bleeding, or necrosis observed around the anastomotic site in the experimental group, which was comparable to the control group (Figure 8). Three-dimensional reconstruction models established from CT image data demonstrated good healing at the anastomotic site in the experimental group, showing no significant differences compared to the control group and in accordance with the experimental design.

**Figure 9:**
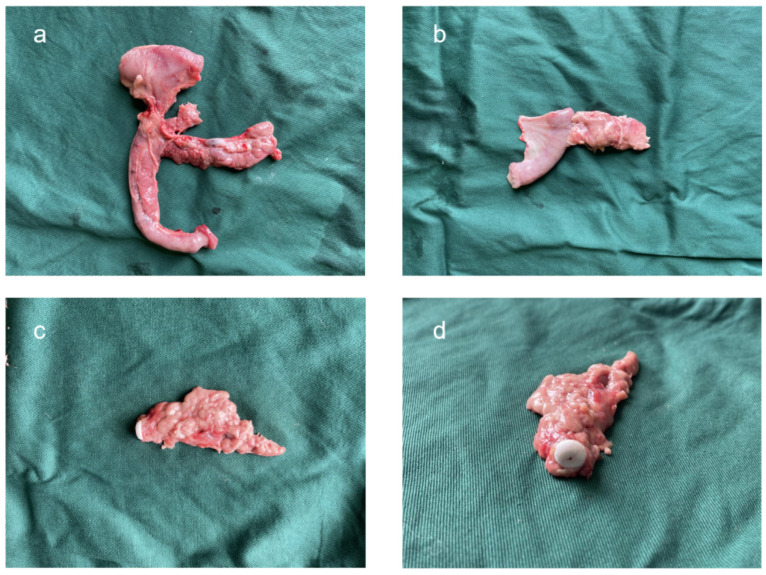
Pancreatic specimens after euthanasia: (**a**) proximal pancreas; (**b**) distal pancreatic body and tail and pancreaticojejunostomy; (**c**) lateral view of the novel stent; (**d**) front view of the novel stent.

After euthanasia, the pancreaticojejunostomy specimens from the experimental group were retrieved and compared with their corresponding 3D reconstruction models. The anastomotic sites in the experimental group, which utilized the novel anastomotic device, showed good healing comparable to the control group (Figure 9). Upon dissection, the anastomotic device was found properly positioned, with no significant changes to the inner surface of the anastomosis, no further dilation of the main pancreatic duct, and unobstructed pancreatic juice drainage. The pancreatic 3D reconstruction was successful, and the 3D-printed models were consistent with the pathological specimens. The anastomotic sites in both the experimental and control groups were observed to have healed well, with mild adhesion to the surrounding tissues and no evident signs of inflammation, edema, abscess, or necrosis, indicating no significant differences between the groups. The control group also showed well-healed anastomoses with mild adhesion to the surrounding tissues, without significant inflammation, edema, abscess, or necrosis.

The 3D-printed pancreaticojejunostomy device demonstrated comparable healing and integration with the surrounding tissue as the traditional manual suturing method, with no significant differences in gross appearance or histological characteristics, suggesting its potential clinical applicability.

## 4. Discussion

The heightened public awareness of health and advancements in diagnostic technology have contributed to an increasing incidence of pancreatic diseases detected annually [14]. This trend has made pancreatic disease research a focal point for surgeons. Pancreaticoduodenectomy (PD) is a critical procedure for tumors in the pancreatic head and periampullary region. Within this procedure, pancreatojejunostomy (PJ) is a key step that significantly influences postoperative complications [15]. The quest to minimize post-PD pancreatic fistulae has driven numerous technical innovations. Despite the variety of techniques available, including pancreaticogastrostomy and various pancreatojejunostomy methods, no single technique has emerged as unequivocally superior [16]. A study by Miron et al. [17] suggested a lower rate of postoperative complications with pancreaticogastrostomy compared to pancreatojejunostomy. However, the choice of technique often relies on the surgeon’s experience, and a definitive comparison of techniques requires a prospective, multicenter, randomized study.

Our study, informed by the expert consensus on postoperative digestive tract reconstruction techniques after pancreatic resection, focused on the duct-to-mucosa pancreaticojejunostomy method. The advent of mechanical anastomosis devices has revolutionized surgical practice, offering simple, fast, and satisfactory anastomosis results [18]. These devices are widely used in gastrointestinal, esophageal, jejunal, and colorectal anastomosis surgeries, simplifying complex procedures. However, they are not exempt from postoperative complications similar to manual suturing, emphasizing the importance of correct device usage.

Accurate pancreatic data acquisition is fundamental to the configuration design of the pancreaticojejunostomy device [19]. The use of imaging techniques, such as CT, MRI, and PET-CT, is crucial for diagnosing pancreatic diseases and differentiating among various pathologies. Multislice helical CT (MSCT), with its rapid scanning, high-quality imaging, and multiphase scanning capabilities, has become an indispensable tool for pancreatic disease assessment [20]. Our study employed MSCT for three-dimensional reconstruction of the pancreas and surrounding structures, implementing standardized protocols to ensure the authenticity and reliability of the imaging data. The manual measurement of three-dimensional organs from two-dimensional images is prone to error, particularly for high-precision devices, such as the pancreatic duct stent. To ensure the accuracy of the reconstruction, our study utilized a multi-observer, multi-directional measurement approach, using the enhanced CT imaging of the portal vein as a reference. This method ensured uniformity in the measurement of the pancreatic neck and duct, enhancing the precision of the reconstruction process.

The development of the pancreaticojejunostomy device is a testament to the integration of advanced materials science and precision surgical techniques. As medical implants, these devices must interact directly with tissues and organs, necessitating materials with excellent biocompatibility, stable mechanical properties, and chemical inertness [21]. This ensures that the material will gradually break down and be absorbed by the body after it has served its purpose, thereby avoiding the need for additional surgical procedures to remove the implant. This is particularly important in surgeries like pancreaticojejunostomy, where the stent is used to support the healing process and maintain the patency of the pancreatic duct. Our choice of polylactic acid (PLA) for the 3D-printed pancreaticojejunostomy device capitalizes on its well-known biocompatibility and biodegradability [22]. PLA is a polymer that has been widely used in various biomedical applications due to its ability to degrade under physiological conditions. The degradation process involves hydrolysis, which breaks down the ester bonds in the PLA polymer chain, eventually reducing it to water, carbon dioxide, and lactic acid, which are naturally metabolized by the body. The thermal analysis of our PLA stent samples, with a start decomposition temperature of 243.90 °C, a peak temperature of 369.33 °C, and an end decomposition temperature of 404.16 °C at a 94.5% decomposition rate, indicates that the material is stable at the average human body temperature of 36–37 °C. This stability is crucial during the initial postoperative period when the stent is providing support to the anastomosis site. However, the exact degradation time of PLA in vivo can vary and is influenced by factors such as molecular weight, crystallinity, and the specific in vivo environment [23]. While current research has not yet reported the precise degradation time for PLA stents in the human body, it is known that PLA can take from several months to over a year to degrade [24]. This timeframe allows for sufficient support during the critical healing phase while ensuring that the material will eventually be reabsorbed as the body’s tissues integrate and strengthen.

The structural integrity of PLA post-3D printing is imperative and was rigorously evaluated through elemental analysis and mechanical property testing. Even with the relatively low thermal stability requirements for biomaterials used in the human body, the processing temperature range stability is crucial for further performance enhancement or molding [25]. Our study ensured that the 3D printing process did not compromise the polymer structure, verified by the lack of significant diffraction angle changes in XRD spectra. Three-dimensional printing technology, with its layer manufacturing and stacking principles, has revolutionized the production of three-dimensional objects. The personalized printing, high repeatability, and rapid prototyping capabilities of 3D printing are unparalleled by traditional manufacturing processes. This technology is particularly advantageous for the production of customized medical implants with high precision requirements and swift production turnaround times. The surface properties of both PLA and silicone stents play a significant role in their interaction with the surrounding tissues. PLA, while more hydrophobic than silicone, was treated to improve its surface characteristics, enhancing cell adhesion and tissue integration. Although silicone’s natural hydrophilicity may promote better tissue integration, the treated PLA in our study demonstrated good biocompatibility with the surrounding tissue. The degradation of PLA into lactic acid, which is metabolized by the body, is a controlled process that does not typically elicit a significant inflammatory response. Our study monitored inflammatory markers and found no substantial difference in the inflammatory response between PLA and silicone stents, suggesting that both materials were well-tolerated in vivo.

Our study employed fused deposition modeling (FDM), a widely used 3D printing technique, to fabricate the pancreaticojejunostomy device from biocompatible PLA. The precision required for the device was stringent, including the design of hollow stent tubes with threaded sections and a supporting disk with specific dimensions to ensure a snug fit with the pancreatic duct and jejunum. The innovative design of our device simplifies the PJ procedure, reducing the number of sutures and minimizing the risk of postoperative complications. The device’s main pancreatic duct stent ensures direct drainage of pancreatic juice into the jejunum, avoiding leakage and bleeding. The supporting disk is designed to match the diameter of the pancreatic neck, pressing the jejunal wall closely against the pancreatic stump and reducing the incidence of fistula.

The use of PLA for the device also addresses the concern of postoperative infection, as the material has shown no increased risk of infection when sterilized with ethylene oxide and handled with aseptic technique during surgery. Furthermore, the material’s application in the experiment did not lead to adverse outcomes such as intestinal obstruction, which is often a concern with surgical implants in animals with complex gastrointestinal anatomy [26,27]. The immunohistochemical analysis from our study indicates that the use of the device did not exacerbate local inflammatory responses, as evidenced by the expressions of IL-6 and IL-10. This finding is crucial, as it suggests that the device does not induce an excessive inflammatory reaction that could hinder the healing process.

While the use of mini pigs in our study provided a valuable model for simulating human pancreatic surgeries due to anatomical and physiological similarities, it is important to acknowledge the limitations when extrapolating these results to human patients. Although 3D printing and the use of PLA material are not new in the field of biomedical engineering, the specific application in pancreaticojejunostomy and the translation to human clinical practice present unique considerations.

Firstly, the porcine pancreatic anatomy, while similar, is not identical to the human pancreas, which may influence the stent’s performance and the healing dynamics at the anastomotic site. Secondly, the metabolic rate and the immunological response in mini pigs could differ from humans, potentially affecting the degradation profile of the PLA stent and the body’s reaction to the material. Additionally, the surgical techniques and postoperative care protocols in a porcine model may not fully replicate those in human surgery, which could also influence the outcomes. It is also important to consider the variability in human patient populations, including differences in age, overall health, and the specific disease context, which could impact the stent’s efficacy and safety. Despite these limitations, our study provides a foundational preclinical evaluation of the 3D-printed PLA stent’s potential in pancreaticojejunostomy. The promising results in mini pigs support the need for further translational research, including clinical trials, to directly assess the stent’s performance in human patients.

In conclusion, while our findings in the mini pig model are encouraging, caution must be exercised when extrapolating these results to humans. Further studies, particularly well-designed clinical trials, are essential to validate the safety and efficacy of the 3D-printed PLA stent in the context of human pancreatic surgery. Precision medicine, underpinned by technologies such as 3D printing, is poised to transform the field of pancreatic surgery. The ability to visualize and simulate surgical procedures preoperatively offers a more intuitive and precise understanding of the patient’s anatomy and the surgical plan. Our study’s preliminary validation of the use of CT-based 3D reconstruction for pancreatic modeling and the development of a 3D-printed biopolymer pancreaticojejunostomy device marks a significant step forward in this direction.

## Figures and Tables

**Table 1:** Comparison of preoperative CT 3D reconstruction measurements and intraoperative actual measurements for pancreatic duct stent design.

Data Measured Based on CT 3D Reconstruction Model before Surgery				Actual Data from Intraoperative Measurements		
No.	Pancreatic Neck Anterior–Posterior Diameter	Pancreatic Neck Superior–Inferior Diameter	Pancreatic Duct Diameter	Pancreatic Neck Anterior–Posterior Diameter	Pancreatic Neck Superior–Inferior Diameter	Pancreatic Duct Diameter
	(mm)	(mm)	(mm)	(mm)	(mm)	(mm)
1	13.9	27	2	13.8	27	1.9
2	15.1	33.2	2.7	15	33.2	2.8
3	21.7	34.5	4.2	21.5	34.4	4
4	14.5	26.3	2.4	14.4	26.4	2.4
5	13.4	30.4	3.2	13.4	30.4	3.1
6	12.8	26	1.9	12.9	26.3	2
7	13.2	29.3	2.1	13.2	29.4	2.1
8	16.6	25.5	3.6	16.5	25.5	3.5
9	13.5	27.2	1.8	13.5	27.2	1.8
10	11.7	32.4	2.3	11.7	32.3	2.4
11	14.6	28.3	2.5	14.6	28.3	2.6
12	13.6	25.9	2.1	13.7	25.8	2.1
13	12.9	28.7	2.2	12.8	29	2.2
14	12.7	27.6	4.1	12.5	27.7	4
15	20.1	32.7	4.7	19.8	33.1	4.6
16	12.4	27.1	1.8	12.7	26.8	1.7
17	14.4	32.4	2.8	14.5	32.4	2.8
18	12.3	26	2.1	12.3	26	2
19	13	29.9	1.9	13.2	30	1.9
20	14.8	31.1	3	14.7	31.5	3.2
21	13.1	26.7	2.7	13.8	26.5	2.7
22	12.5	30.1	3.3	12.4	30	3.1
23	15.5	25	2	15.4	25	2
24	13.7	32.6	2.2	13.7	32.6	2.1
25	17.1	24.7	2.1	17.3	24.5	1.9
26	13.8	27.5	1.7	13.8	27.2	1.7
27	12.5	33	2.3	12.7	33.2	2.1
28	13.2	32.4	4.2	13.2	32.6	4.2
29	12	24.2	2.8	12	24.2	2.7
30	11.4	33.5	2.1	11.3	33.2	2.1
31	15.3	32.1	3	15.3	32	2.8
32	14.3	34.8	2.5	14.4	34.8	2.5

**Table 2:** Postoperative complications.

Postoperative Complications	Experimental Group	Control Group
Pancreatic fistula	2	2
Intestinal obstruction	0	0
Peritonitis	0	1
Postoperative hemorrhage	0	1
Acute pancreatitis	1	1
Death	0	1

**Table 3:** Surgical parameters between experimental group and control group.

Parameter	Experimental Group	Control Group	*p*
Second surgery time (min)	105.12 ± 15.57	123.62 ± 11.84	<0.001
Pancreaticojejunostomy time (min)	43.01 ± 10.61	57.75 ± 8.71	<0.001
Anesthesia recovery time (min)	39.25 ± 12.72	30.50 ± 8.20	<0.001
Intraoperative blood loss (mL)	32.125 ± 12.10	25.75 ± 6.48	<0.001
Time to start eating post-op (h)	within 24 h	within 24 h	0.674

**Table 4:** Comparison of blood factor concentrations 2 months after pancreaticojejunostomy.

Parameter	Experimental Group	Control Group	*p*
Serum C reactive protein (CRP)	<0.02	<0.02	0.174
Procalcitonin (PCT)	<0.5	<0.5	0.681
IL-6	3.057 ± 0.122	3.052 ± 0.110	0.291
IL-10	10.470 ± 0.784	9.894 ± 0.573	0.284

## Data Availability

The data that support the findings of this study are available from the corresponding author, F.L. upon reasonable request.
